# A negative piezo-conductive effect from doped semiconducting polymer thin films

**DOI:** 10.1038/s41598-021-97812-4

**Published:** 2021-09-14

**Authors:** Chao Yi, Lening Shen, Jie Zheng, Xiong Gong

**Affiliations:** 1 School of Polymer Science and Polymer Engineering, The University of Akron, Akron, OH 44325 USA; 2 Department of Chemical, Biomolecular and Corrosion Engineering, College of Engineering and Polymer Science, The University of Akron, Akron, OH 44325 USA

**Keywords:** Materials science, Nanoscience and technology, Physics

## Abstract

In the past years, piezo-conductive sensors have drawn great attention in both academic and industrial sectors. The piezo-conductive sensors made by inorganic semiconductors exhibited poor mechanical flexibility, restricting their further practical applications. In this study, we report the piezo-conductive sensors by a semiconducting polymer, poly(3,4-ethylenedioxythiophene) doped with tosylate ions (PEDOT:Tos) thin films. Systemically studies indicate that the piezo-conductive response of the PEDOT:Tos thin films is originated from the deformation of the PEDOT crystal cells and the stretched π–π distances induced by Tos. Moreover, the negative piezo-conductive effect, for the first time, is observed from PEDOT:Tos thin film under the pressure. A working mechanism is further proposed to interpret the transient from a positive to a negative piezo-conductive response within the PEDOT:Tos thin films. Our studies offer a facile route to approach effective piezo-conductive sensors based on conjugated polymers.

## Introduction

Piezo-conductive sensors are widely utilized in construction, automotive, computer, medical and homeland sectors^1–4^. Currently, most of the piezo-conductive sensors were made by inorganic semiconductors^1–8^. However, these inorganic-based sensors were typically fabricated by a cost-ineffective top-down lithographic technique^5–8^, and possessed poor mechanical flexibility, which was ascribed to their rigid properties^9^. To overcome these problems, piezo-conductive sensors based on polymer-carbon nanotube (CNTs) composites were developed^10,11^. In these stratagems, insulating polymers were used as the matrix to provide flexibility, and CNTs were used as the electrical conduction channels. These novel piezo-conductive sensors exhibited flexibility but possessed poor reliability due to their anisotropic piezo-conductive responses^10,11^. Moreover, due to the difficulties in forming uniformly distributed CNTs and challenges in aligning CNTs in the polymer matrix, the responses of these piezo-conductive sensors were extremely poor under either stress or pressure^10,11^.

Semiconducting polymers, which possess high flexibility, uniform film quality, tunable electrical properties, and low-cost fabrication processability, are complementary alternatives for making new types of piezo-conductive sensors^12–14^. It was reported that the piezo-conductive sensors based on poly(3,4-ethylenedioxythiophene):polystyrene sulfonate (PEDOT:PSS) thin film exhibited good flexibility, air-stability, higher sensitivity, and smaller relaxation time^13–15^. But, the applications of the piezo-conductive sensors based on PEDOT:PSS thin films were restricted by their low electrical conductivity, which was originated from PSS insulator, and its amorphous structure as the randomly packed PEDOT chains in the PEDOT:PSS thin film^16–18^.

In this study, we report the piezo-conductive sensors fabricated by a semiconducting polymer, poly(3,4-ethylenedioxythiophene) doped with tosylate ions (PEDOT:Tos) thin films. Systemically studies indicate that the piezo-conductive response of the PEDOT:Tos thin films is originated from the deformation of the PEDOT crystal cells and the stretched π-π distances induced by Tos. Moreover, the negative piezo-conductive effect, for the first time, is observed from PEDOT:Tos thin film under the pressure. A working mechanism is further proposed to interpret the transient from a positive to a negative piezo-conductive response from PEDOT:Tos thin films.

## Results and discussion

PEDOT was a typical conducting polymer, which was widely used in electronic and optoelectronics, electrochromic displays, printed wiring, and sensors^19,20^. However, PEDOT thin films typically possessed poor electrical conductivities (9 × 10^–4^ S/cm), which restricted its applications^19,20^. To enhance the electrical conductivity of PEDOT thin films, in this study, the small-sized Tos ions are used to dope PEDOT to create PEDOT:Tos thin films. The preparation procedures of PEDOT:Tos thin films are described in the Experimental Section.

**Figure 1:**
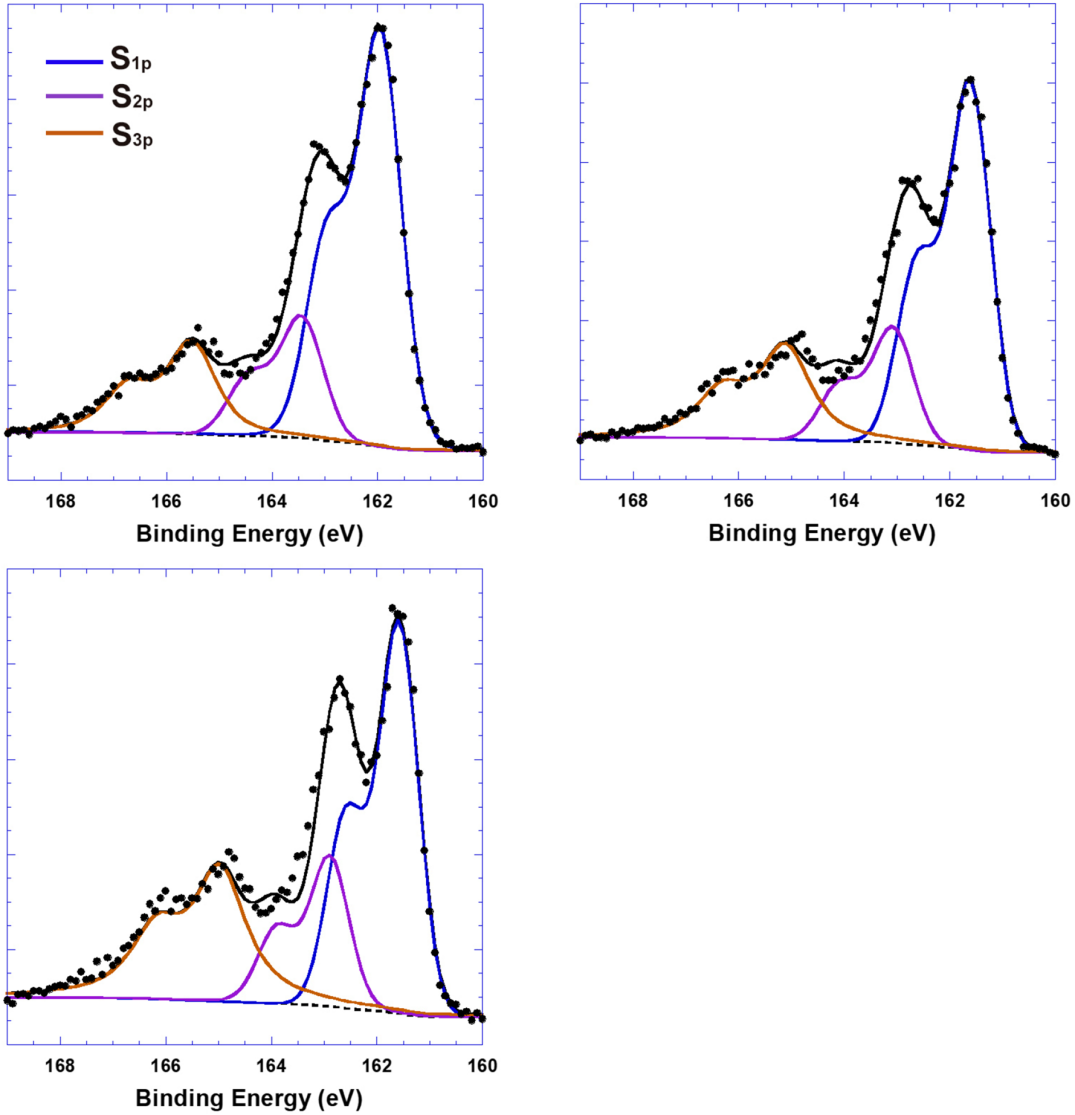
The fitted S_2p_ XPS spectra of PEDOT:Tos thin films with the doping levels of Tos at (**a**) 23.7%, (**b**) 31.6%, and (**c**) 43.6%.

X-ray photoelectron spectroscopy (XPS) is first carried out to confirm that Tos is indeed doped into PEDOT. The fitting procedure of XPS is described in Supporting Information (SI) 1. Figure 1 presents the XPS spectra of PEDOT:Tos thin films. The spin-split core levels of the sulfur (S) in the PEDOT:Tos chain is located in the binding energy ranging from 160 to 168 eV^21,22^. The binding energies of S in the PEDOT:Tos thin films with different concentrations of Tos ions are dramatically different, which confirms that Tos is indeed doped into PEDOT thin films^21,22^. Moreover, the doping levels of Tos in the PEDOT:Tos thin films can be calculated based on the integrated area under the fitted curves in XPS spectra. The S in PEDOT chains of pristine PEDOT thin films is labeled as *S*^*2*^* p*_*1/2*_ and *S*^*2*^* p*_*3/2*_, the S in the PEDOT chains of the PEDOT:Tos thin films are labeled as *S*^*1*^* p*_*1/2*_ and *S*^*1*^* p*_*3/2*_, and the spin-spilled signals from sulfonate group in Tos^-^ is labeled as *S*^*3*^*p*. The spin-spilled peak to peak distance is set to be 1.0 eV and the full width at half maximum is set to be 0.85 eV. The ratios between Tos^-^ ions and S in PEDOT chains are obtained through the calculation of the integrated area under the fitted curves. Thus, the doping levels of Tos (by molar ratio) in the PEDOT:Tos thin films are calculated to be 23.7%; 31.6%; and 43.6%, respectively.

**Figure 2:**
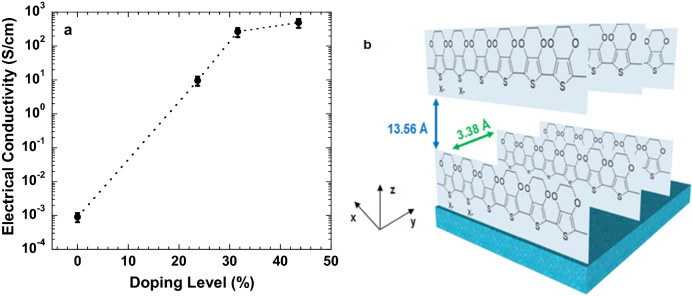
(**a**) The electrical conductivities of the PEDOT:Tos thin films with different doping levels of Tos; and (**b**) the proposed molecular packing of the PEDOT:Toss thin film (at the doping level of 43.6%).

Figure 2a presents the electrical conductivities of the PEDOT:Tos thin films versus the doping levels of Tos ions. The electrical conductivity of pristine PEDOT is ~ 9 × 10^–4^ S/cm^19,20^. It is found that the electrical conductivities of the PEDOT:Tos thin films are increased along with the increased doping levels of Tos ions. The optimal electrical conductivity of 493 S/cm is observed from the PEDOT:Tos thin film with a doping level of 43.6%. However, further increasing the doping levels of Tos cannot be realized due to the restricted polymerization in the thin film preparation^17^.

As indicated in Figure 2b, the electrical conductivities of PEDOT:Tos thin films are depended on the inter-chain distance (d_z_) and the π-π distance, and both of them are related to the PEDOT chain on the perpendicular and parallel directions^18,23^. The perpendicular direction is the set of the Bragg peaks along the out-of-plane direction (q_z_), and the parallel direction is the set of the Bragg peaks along the in-plane direction (q_y_). In the q_z_ direction, charge transport is along the long-ranged, ordered, and lateral packing of the PEDOT backbones (d_z_), while, in the q_y_ direction, charge transport is through the π-π stacking of the thiophene rings^24^.

**Figure 3:**
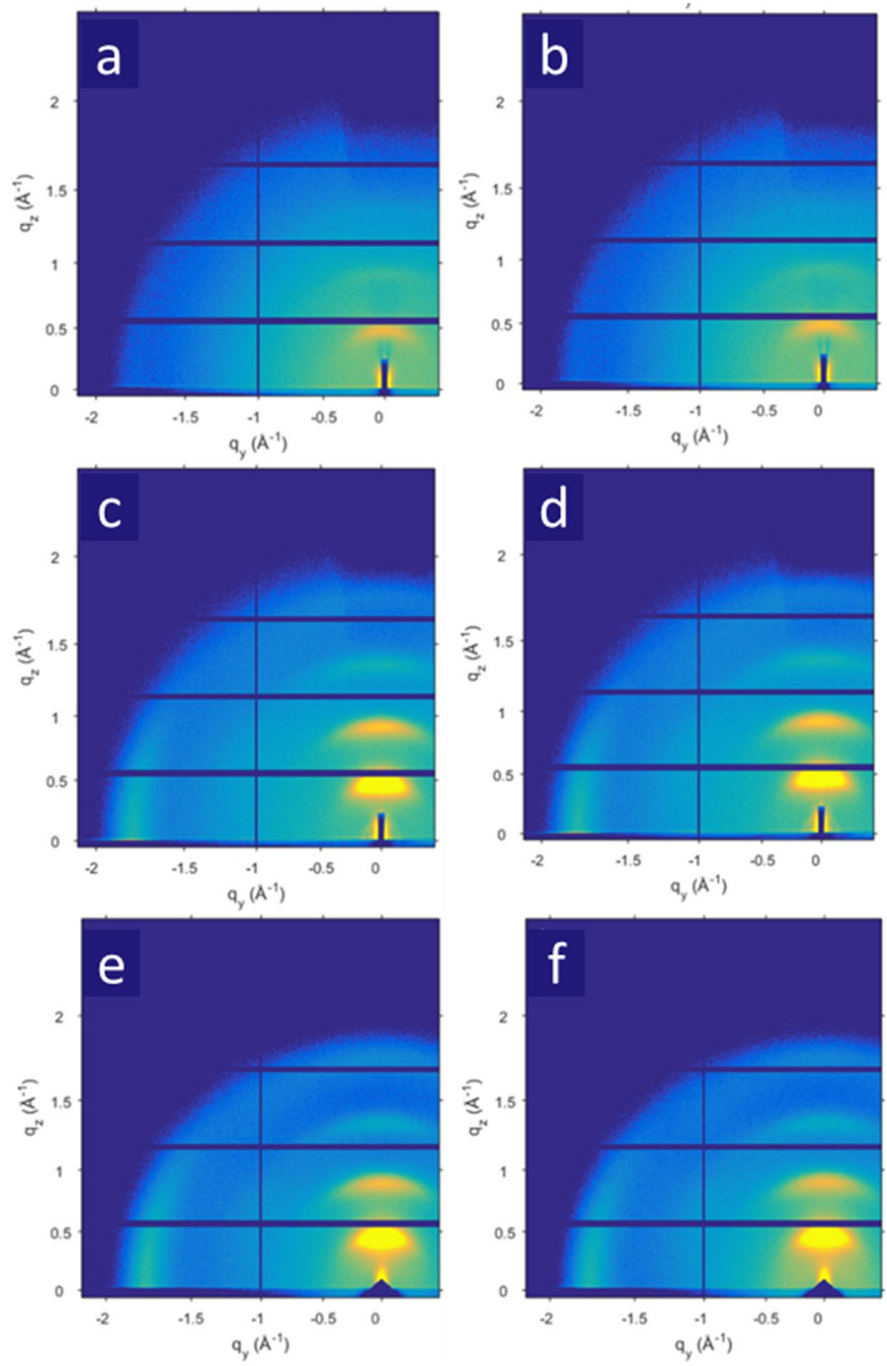
The GIWAXS patterns of the PEDOT:Tos thin films with different doping levels of Tos at (**a**) 23.7%; (**c**) 31.5%; and (**e**) 43.6% under no nitrogen flow pressure; and (**b**) 23.7%; (**d**) 31.5%; and (**f**) 43.6% under the nitrogen flow pressure.

**Figure 4:**
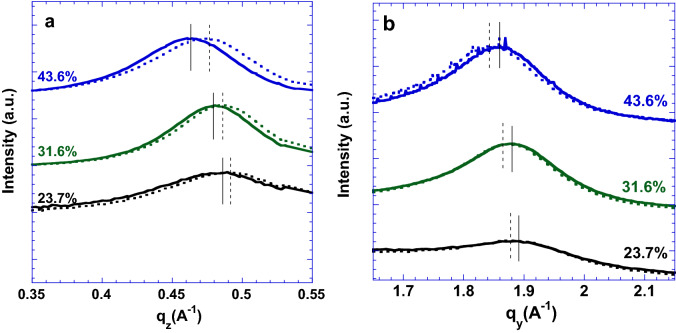
The 1D GIWAXS patterns along (**a**) q_z_, and (**b**) q_y_ of the PEDOT:Tos thin films at the doping levels of 23.7%, 31.6%, and 43.6% under the nitrogen flow pressure (dash line) and under no nitrogen flow pressure (solid line).

The grazing incidence wide-angle X-ray scattering (GIWAXS) is carried out to understand underlying enhanced electrical conductivities of the PEDOT:Tos thin films. Figure 3 displays the GIWAXS patterns of the PEDOT:Tos thin films with different doping levels of Tos under nitrogen flow pressure and no nitrogen floe pressure. Both the set of the Bragg peaks along the out-of-plane and the in-plane directions are identified. The positions of the Bragg peaks along the out-of-plane and the in-plane directions represent the inter-chain spacing between adjacent PEDOT chains and the π-π distance between one PEDOT chain faced another PEDOT chain. Based on Figure 3, the one-dimension (1D) GIWAXS patterns of the PEDOT:Tos thin films are obtained and the results are shown in Figure 4a, b. Table 1 summarizes q_z_, q_y_, the inter-chain distance (d_z_), and the π-π distance of the PEDOT:Tos thin films. It is found that the π-π distances of the PEDOT:Tos thin films are increased along with increased doping levels. Such increased π-π distance is a result of the accumulation of the counter-ions. These counter-ions could coordinate with the PEDOT chain and occupy a larger volume, resulting in both increased d_z_ and the π-π distances. In addition, the charge carrier concentrations are increased along with increased doping levels, resulting in increased electrical conductivities. On the other hand, it is found that the d_z_ of the PEDOT:Tos thin films are increased along with increased doping levels. Such increased d_z_ indicates that the long-ranged, ordered and lateral packing of the PEDOT backbones are pronounced in the PEDOT:Tos thin films. All these results demonstrate that the electrical conductivities of the PEDOT:Tos thin films are enhanced as the doping levels of Tos are increased.

**Table 1:** Structural parameters of the PEDOT:Tos thin films.

Doping level (%)	q_z_ (Å^−1^)	d_z_ (Å)	q_y_ (Å^−1^)	π–π distance (Å)
23.6	0.4861 (0.4916)	12.926 (12.781)	1.8875 (1.8811)	3.329 (3.340)
31.6	0.4794 (0.4861)	13.106 (12.926)	1.8833 (1.8694)	3.336 (3.361)
43.6	0.4633 (0.4767)	13.562 (13.181)	1.8597 (1.8430)	3.379 (3.409)

*The data present in the parentheses are those under nitrogen flow pressure.

**Figure 5:**
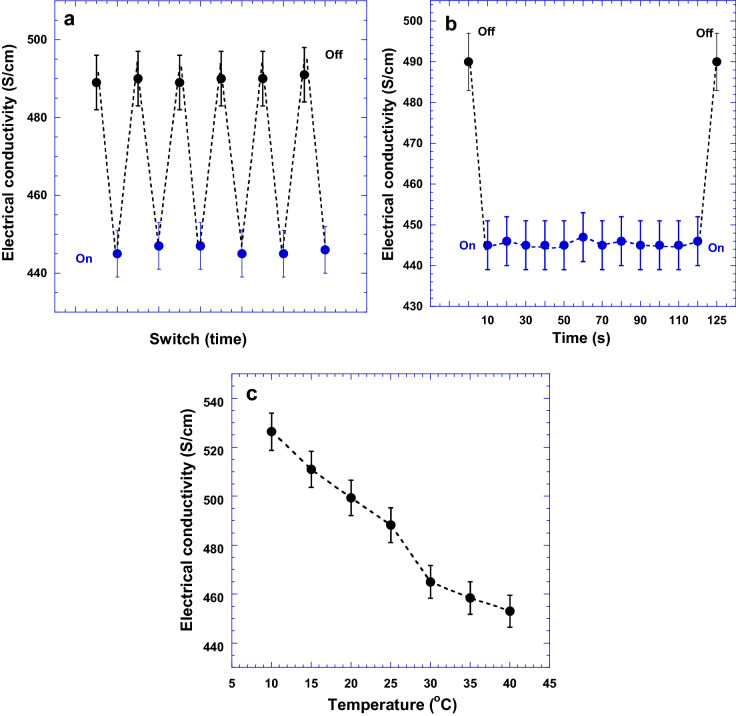
The electrical conductivities of the PEDOT:Tos thin films with the doping level of 43.6% (**a**) under the nitrogen flow pressure and under no nitrogen flow pressure, (**b**) under different flow times, and (**c**) under different temperatures without nitrogen flow pressure.

Figure 5a presents the electrical conductivities of the PEDOT:Tos thin film with a doping level of 43.6% as it is under the nitrogen flow pressure and no nitrogen flow pressure as well. The electrical conductivity of the PEDOT:Tos thin film is measured to be 490 ± 7 S/cm, whereas the electrical conductivity is reduced to 445 ± 6 S/cm as the thin film is under the nitrogen flow pressure for 1 s (s). Remarkably, the reduced electrical conductivity is immediately recovered to its original value once the nitrogen flow pressure is released. The electrical conductivities of the PEDOT:Tos thin films with other doping levels under the nitrogen flow pressure are presented in SI 2. A similar phenomenon is also observed from the PEDOT:Tos thin films with other doping levels. All these results indicate that electrical conductivities of the PEDOT:Tos thin films are affected by the nitrogen flow pressure.

Figure 5b presents the changes of electrical conductivities of the PEDOT:Tos thin film as it is under the nitrogen flow pressure for 120 s, and after the nitrogen flow pressure is released. The electrical conductivities remain at reduced values as the PEDOT:Tos thin films are under the nitrogen flow pressure, but they are instantly recovered to their original values once the nitrogen flow pressure is released.

In order to exclude the temperature effect on the electrical conductivities of the PEDOT:Tos thin films under the nitrogen flow pressure, the electrical conductivities of the PEDOT:Tos thin film under different temperatures are studied and the results are shown in Figure 5c. The electrical conductivities of the PEDOT:Tos thin films are decreased along with increased temperatures. Thus, the temperature variation has no effects on the decreased electrical conductivities of the PEDOT:Tos thin film under the nitrogen flow pressure.

The q_z_, q_y_, the inter-chain distance (d_z_), and the π-π distance of the PEDOT:Tos thin films under the nitrogen flow pressure are summarized in Table 1. As compared with the PEDOT:Tos thin films under no nitrogen flow pressure, decreased d_z_ values are observed from the PEDOT:Tos thin films under the nitrogen flow pressure. These results indicate that the packing of the PEDOT backbones is relatively disordered, resulting in reduced electrical conductivity^18,23^.

**Figure 6:**
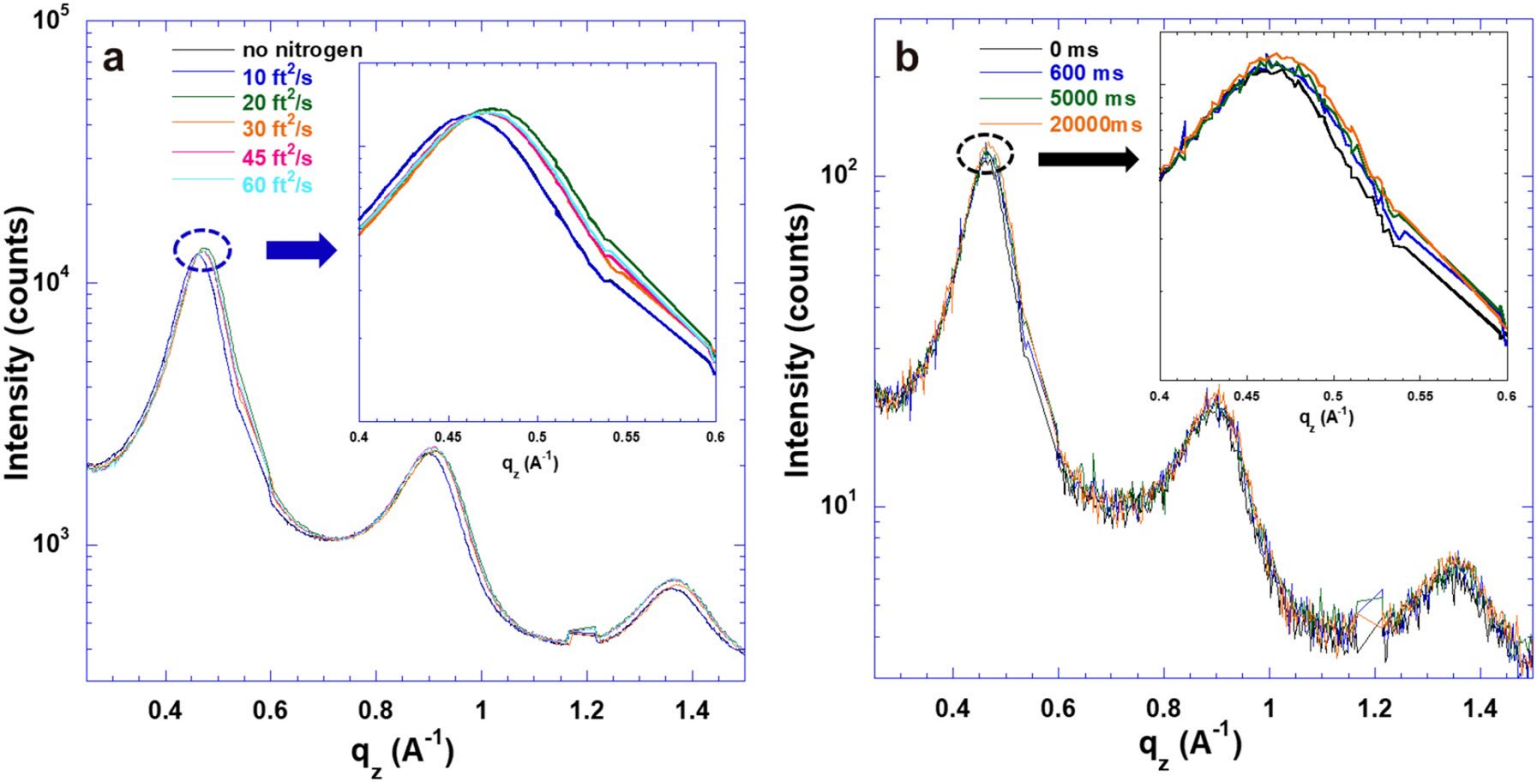
The 1D-GIWAXS patterns of the PEDOT:Tos thin film, (**a**) under different nitrogen flow rates, and (**b**) under helium with the flow rate of 20 ft^2^/s for different times.

To further understand how the nitrogen flow pressure affects the film morphologies at nanoscale, the electrical conductivities of the PEDOT:Tos thin films under different flow rates are investigated. Figure 6a shows the 1D GIWAXS patterns of the PEDOT:Tos thin film along the out-of-plane direction under the nitrogen flow with different flow rates. The Bragg peaks are shifted to a higher q_z_ value as the thin films are under the nitrogen flow with the flow rate up to 30 ft^2^/s and no further increase in q_z_ is observed as the gas flow rate is over 30 ft^2^/s. These results indicate that the PEDOT inter-chain spacing is fully compressed as the thin film is exposed to the gas flow over 30 ft^2^/s and further demonstrate the gas flow rate (or pressure) has great influences on the deformation of the PEDOT crystal cells.

Figure 6b displays the positions of the Bragg peaks along the out-of-plane direction of the PEDOT:Tos thin films under the gas pressure with a flow rate of 20 ft^2^/s at different times. A distinguishable shift of the Bragg peak is observed as the thin film is under the nitrogen flow after 600 ms (ms). No further Bragg peak shift is observed even though the thin film is under the nitrogen gas flow for over 20,000 ms. These results indicate that the deformation of the PEDOT crystal cells is a transient process that takes place within 0.6 s. Such short transient time and stable deformation of the PEDOT crystal cells are responsible for the fast and stable changes in the electrical conductivities.

**Figure 7:**
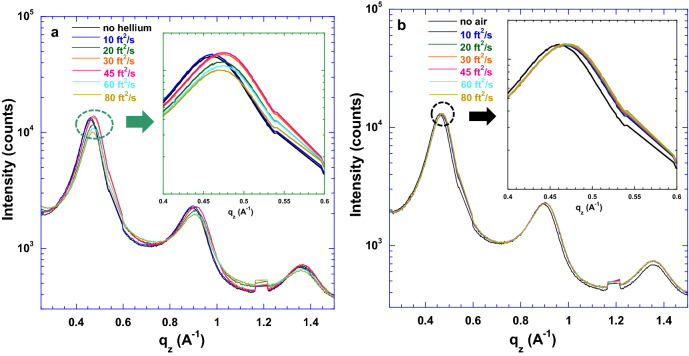
The 1D-GIWAXS patterns of the PEDOT:Tos thin film with the doping level of 43.6% under (**a**) helium, (**b**) compressed air flow pressure with various flow rates.

The PEDOT:Tos thin films exposed to helium with different flow rates are conducted for the verification of the deformation of the PEDOT crystal cells affected by the gas flow rate rather than the gas itself. The 1D GIWAXS patterns of the PEDOT:Tos thin film along the out-of-plane direction under different gas flow with different flow rates are shown in Figure 7. A similar phenomenon is observed from the PEDOT:Tos thin films exposed to different gases flow with different flow rates, which demonstrates that the piezo-conductive effect of the PEDOT:Tos thin films are affected by the gas flow rates rather than gas itself. Moreover, these results indicate that the PEDOT:Tos thin films have potentials applications for in-situ gas flow rate (pressure) monitoring, dynamic mechanical stress, and strain recording^1–4^.

**Figure 8:**
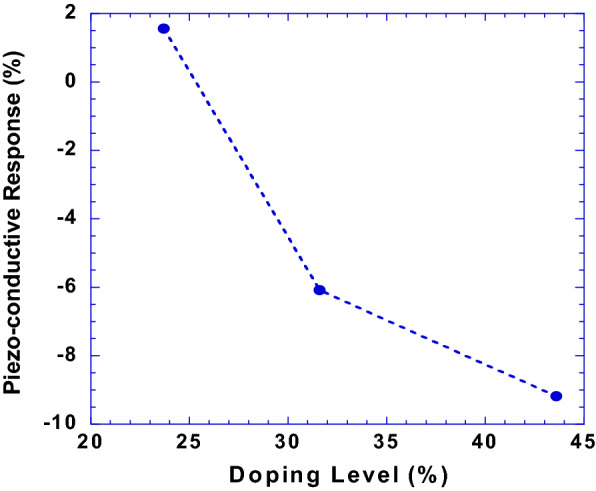
The piezo-conductive responses of the PEDOT:Tos thin films with different doping levels of Tos.

To explore the PEDOT:Tos thin films in the applications of piezo-conductive sensors, the piezo-conductive responses (*R*), which is defined as $R = \frac{{\sigma_{off} - \sigma_{on} }}{{\sigma_{off} }}$, where σ_off_ and σ_on_ are the electrical conductivities of the PEDOT:Tos thin films under no gas flow pressure and gas flow pressure, respectively, are investigated^25–27^. Figure 8 presents the piezo-conductive responses (*R*) of the PEDOT:Tos thin films versus the doping levels of Tos. The *R* values are decreased along with increased doping levels of Tos. It is found that as the doping level of Tos is larger than 27%, the *R* is smaller than zero, which indicates that the PEDOT:Tos thin films possess a negative piezo-conductive effect. To the best of our knowledge, such an “abnormal phenomenon” is the first time observed from piezo-conductive sensors^4,28,29^.

**Scheme 1:**
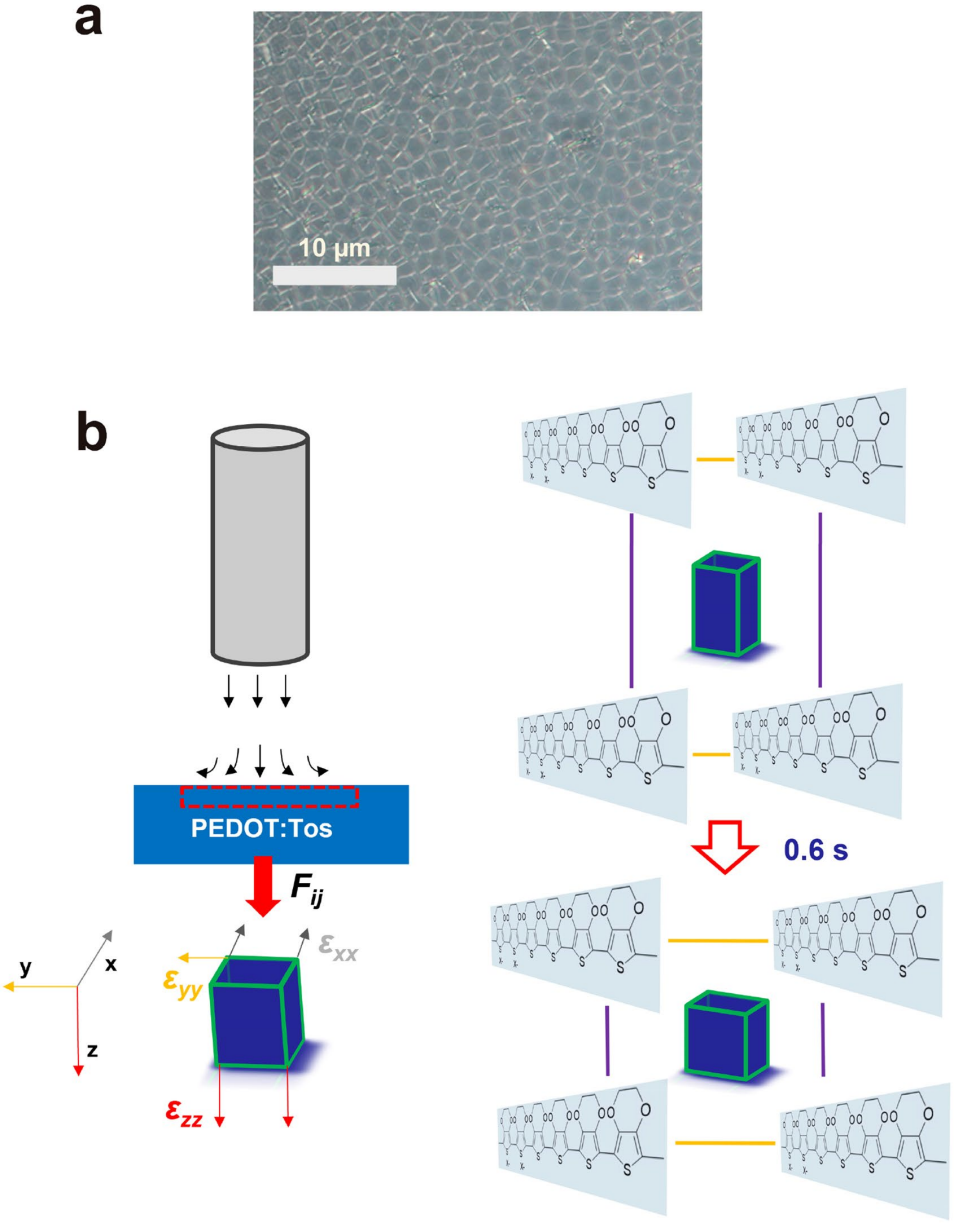
(**a**) Optical spectroscopy of the PEDOT:Tos thin film with the doping level of 43.6%; (**b**) the proposed deformation of the PEDOT crystal cell under the gas flow pressure.

To better understand the piezo-conductive effects in the PEDOT:Tos thin film, a proposed model of the deformation of the PEDOT crystal cells under the gas flow pressure is shown in Scheme 1. This proposed model is based on the crystalline regions as shown in Scheme 1a and the GIWAXS patterns. It is speculated that the PEDOT crystal cells within the PEDOT:Tos thin films possess a rectangular geometry. As the PEDOT:Tos thin film is under the gas flow pressures, the pressure induces a compressive force on the rectangular PEDOT crystal cells, resulting in the PEDOT crystal cells with a geometric deformation. The compressive force could be represented by a force tensor, *F*_*ij*_. Thus, the deformation of the PEDOT crystal cell could be described by a strain tensor, which is defined as $\varepsilon = \frac{1}{2}\left( {\nabla_{{\varvec{A}}} {\varvec{F}} + \left( {\nabla_{{\varvec{A}}} {\varvec{F}}} \right)^{T} + \left( {\nabla_{{\varvec{A}}} {\varvec{F}}} \right)^{T} \cdot \nabla_{{\varvec{A}}} {\varvec{F}}} \right)$, where ***F*** is the force tensor, ***A*** is the area tensor. Let us assume the PEDOT chain is a rigid backbone, thus, it will not be rotated or be compressed as it is under the compressive force. Therefore, the above equation can be simplified, and the strains are only composed of three factors, $\varepsilon_{xx} = F_{xx} /A_{xx} \cdot E$, along the PEDOT chains (where E is the modulus of the PEDOT chain), $\varepsilon_{yy} = F_{yy} /A_{yy} \cdot E_{interaction}$, along the π-π direction between adjacent PEDOT chains, (where $E_{interaction}$ is determined by the interaction between the thiophene face-to-face electron conjugation) and $\varepsilon_{zz} = F_{zz} /A_{zz} \cdot E_{packing}$, along the out-of-plane direction (where $E_{packing}$ is defined by the packing of the PEDOT chains). Under the strain, $\varepsilon_{xx}$, the PEDOT backbone is stretched, thus, the doped counter-ions will be re-distributed along with the PEDOT repeat units, which could modify the doping states of the thin film. Since this strain is strongly dependent on the modulus of the PEDOT chain, E, thus, the molecular weight of PEDOT will have remarkable influences on the deformation of the PEDOT crystal cells. For the strain $\varepsilon_{yy}$ along with the π-π distance, it is correlated with the interaction modulus of the PEDOT conjugation, $E_{interaction}$, thus, the thermal fluctuation and the doping level of the PEDOT:Tos thin film would have great impacts on the deformation of the PEDOT crystal cells. For the $\varepsilon_{zz}$, as it is determined by the molecular arrangement of the PEDOT chains, $E_{packing}$, the packing stratagem of the PEDOT chains would also affect the deformation of the PEDOT crystal cells.

Based on the above model and the GIWAXS results, the strain of the PEDOT crystal cells can be estimated. For example, the PEDOT crystal cells within the PEDOT:Tos thin film with a doping level of 43.6% possess a strain along the pressure direction, $\varepsilon_{zz} = - \frac{{\Delta d_{z} }}{{d_{z} }} = 2.80 \%$. Thus, we assume that the strain, $\varepsilon_{zz}$ of the PEDOT crystal cells represent the strain of the thin film under the gas flow pressure. With increased doping levels, the R is decreased and finally converted into a negative value, which was previously solely observed in n-type inorganic semiconductors^15,25,26^. Therefore, the negative piezo-conductive effect is observed from the PEDOT:Tos thin films.

In summary, for the first time, we reported doping-induced transition from the positive to the negative piezo-conductive effects in the highly conductive crystalline tosylate ions (Tos) doped semiconducting polymer, poly(3,4-ethylenedioxythiophene) (PEDOT) thin films. It was found that the electrical conductivities of PEDOT:Tos thin films were increased along with increased doping levels of Tos, which was originated from the elongated PEDOT backbones and enlarged π-π distance of thiophene rings. Further studies demonstrated that the piezo-conductive responses of the PEDOT:Tos thin films are ascribed to the deformation of the PEDOT crystal cells. Moreover, studies illustrated that the stretched π–π distances were responsible for the decreased piezo-conductive responses and the transition from the positive to the negative piezo-conductive responses. We further proposed a model and discussed the possible factors that could influence the deformation of the PEDOT crystal cells. Our studies offered a facile route to approach effective piezo-conductive sensors based on conjugated polymers.

## Experimental

### Materials

Ferric tosylate (Fe(tos)_3_) was prepared according to the reported method. The ethylenedioxythiophene (EDOT, 97%) monomer was purchased from Alfa Aesar. Pyridine and n-butanol (anhydrous, 99%) were purchased from Sigma-Aldrich. All materials are used as received without further purification.

### Preparation of PEDOT:Tos thin films

Pyridine (13.8 µL) was added into Fe(tos)_3_ (0.25 g, 0.30 g or 0.40 g) mixed with 0.6 g n-butanol solution and then stirred at room temperature for 12 h (hrs). Afterward, the EDOT monomer (34 µL) was added into the above solution at room temperature to form PEDOT:Tos precursor solution. After that, PEDOT:Tos thin films were deposited on pre-cleaned glass substrates from above precursor solution by spin coating, followed thermal annealed at 70 °C for 1 h.

### Characterizations of PEDOT:Tos thin films

The thickness of PEDOT:Tos thin films were measured by Dektak 150 surface profilometer with a scan rate of 0.06 mm/s and further confirmed by the X-ray reflectivity (XRR) (Figure S2). The PEDOT:Tos thin film at a doping level of 43.6% presents a constant film thickness ~ 219.5 nm as it is under the nitrogen flow pressure and no nitrogen flow pressure. X-ray photoelectron spectroscopy (XPS) spectra were obtained by a PHI 5000 Versa Probe II scanning XPS microprobe to identify the chemical components in the PEDOT:Tos thin films. For electrical conductivity measurement, aluminum (Al) electrode (with a thickness ~ 150 nm) was deposited atop the 4 corners of the PEDOT:Tos thin films through a shadow mask under a vacuum with a base pressure of 2 × 10^–4^ Pa. Van der Pauw four-point probe method was used to measure the electrical conductivities of the thin films. The distance between Al electrodes is measured to be 15 mm. Note that for the measurement of the electrical conductivities of the PEDOT:Tos thin films under the nitrogen gas flow pressure, the nitrogen gas had a flow rate of 60 ft^2^/s, and the distance between the thin films and the exit of the tube was 1 cm. Grazing incidence wide-angle X-ray scattering (GIWAXs) patterns of the PEDOT:Tos thin films were measured in beamline 8-ID-E, Advanced Photon Source, Argonne National Laboratory. The incident angle is 0.14°. The GIWAXS data were collected by exposing the samples in X-ray for 10 s and the collected data were analyzed by using Matlab-based software (GIXSGUI). The optical microscopy images of the PEDOT:Tos thin film were captured by a digital camera (EOS 5D2, Canon) on POM (BX60, Olympus) optical microscopy.

## Supplementary Information

Supplementary Information.
