# Supplementary material for: A negative piezo-conductive effect from doped semiconducting polymer thin films
Source: Sci Rep. 2021 Sep 14;11:18222. doi: 10.1038/s41598-021-97812-4 (PMC8440650; doi:10.1038/s41598-021-97812-4)
Supplement: Supplementary file 1 — Supplementary Information. [file 41598_2021_97812_MOESM1_ESM.docx]

**Supporting information for**

**A negative piezo-conductive effect from doped semiconducting polymer thin films**

Chao Yi,^1,#^ Lening Shen,^1,#^ Jie Zheng^2^ and Xiong Gong*^,1^

^1)^ School of Polymer Science and Polymer Engineering, and ^2)^ Department of Chemical, Biomolecular and Corrosion Engineering, College of Engineering and Polymer Science, The University of Akron, Akron, OH, 44325

**SI 1 Fitting XPS spectroscopes**

**SI 2 The electrical conductivity of the PEDOT:Tos thin films under the nitrogen flow pressure**

**SI 3 The 1D GIWAXS patterns of the PEDOT:Tos thin films along the out-of-plane direction under different gases flow with different flow rates**

**SI 4 X-ray reflectivity of the PEDOT:Tos thin films**

# These authors have contributed to this work equally.

* Corresponding author, E-mail: [xgong@uakron.edu](mailto:xgong@uakron.edu); Fax: (330) 972-3406

**SI 1 Fitting XPS spectroscopes**

Three different types of sulfur elements were identified. One is from the PEDOT chain that is not doped by Tos as the doping level does not reach 100%. The second is that from PEDOT doped by Tos. The third sulfur then is assigned to that in Tos as its chemical environment is different from sulfur in PEDOT chains. The positions of the assigned sulfur elements were compared with data in the PHI (the manufacture of the facility) XPS handbook to make sure they locate within the ranges that had been reported in the handbook. Fitting XPS spectra of the PEDOT:Tos thin films at different doping levels are processed on XPSPEAK41 developed by Dr. Raymund W.M. Kwok in the Department of Chemistry, The Chinese University of Hong Kong. The fitting processes are under the following routes: the spin-split core levels feature originating from the sulfurs (S^*^) in the PEDOT chain have peaked from 160-168 eV, then, the contributions from S^*^ in the undoped PEDOT chains are labeled as S^1^ p, which have spin-spit signals from S^1^ p_1/2_ and S^1^ p_3/2_. The signals from S^*^ in the doped PEDOT chains are labeled as S^2^ p, which also has spin-spit signals from S^2^ p_1/2_ and S^2^ p_3/2_. The spin-spilled peak to peak distance (s. o. s) is set to 1.0 eV and the full width at half maximum (FWHM) is set to 0.85 eV. The spin-spilled signals from the sulfonate group in Tos^-^ are labeled as S^3^p. By calculating the area under the fitted curves from the above-assigned elements, the ratios between Tos^-^ counter-ions and S^*^ in the PEDOT chains could be calculated. Then the doping level of the PEDOT chain could be estimated by these ratios.

**SI 2 The electrical conductivity of the PEDOT:Tos thin films under the nitrogen flow pressure**

**Figure S1.** The electrical conductivities of PEDOT:Tos thin films at the doping levels of a) 23.7%; b) 31.5%; and c) 43.6% under the nitrogen flow pressure (on) and under no nitrogen flow pressure (off).

**SI 3 X-ray reflectivity of the PEDOT:Tos thin films**

**Figure S2** shows the X-ray reflectivity (XRR) of the PEDOT:Tos thin film under the nitrogen flow pressure and no nitrogen flow pressure. From the maximum between two adjacent reflectivities, the thickness of the thin film can be calculated: $d=\frac{\lambda}{2}\cdot\frac{1}{\Delta\theta}$, where d is the film thickness, λ is the wavelength of the X-ray, ∆θ is the different theta between the maximums of two adjacent reflectivities. The calculated thickness of the PEDOT:Tos thin film with and without nitrogen flow pressure is the same, 219.5 nm.

**Figure S2**. X-ray reflectivities of the PEDOT:Tos thin film at the doping level of 43.6% under the nitrogen flow pressure and under no the nitrogen flow pressure.
